# Metal Oxide vs Organic Semiconductor Charge Extraction Layers for Halide Perovskite Indoor Photovoltaics

**DOI:** 10.1002/smsc.202400292

**Published:** 2024-09-10

**Authors:** Shaoyang Wang, Tim Kodalle, Sam Millar, Carolin M. Sutter‐Fella, Lethy Krishnan Jagadamma

**Affiliations:** ^1^ Energy Harvesting Research Group, School of Physics & Astronomy, SUPA University of St Andrews North Haugh, St Andrews Fife KY16 9SS UK; ^2^ Molecular Foundry Lawrence Berkeley National Laboratory 1 Cyclotron Road Berkeley CA 94720 USA; ^3^ Advanced Light Source Lawrence Berkeley National Laboratory 1 Cyclotron Road Berkeley CA 94720 USA

**Keywords:** grazing incidence wide-angle X-Ray scattering, hole extraction layers, indoor light harvesting, interface passivation, light soaking

## Abstract

Halide perovskite indoor photovoltaics (PVs) are highly promising to autonomously power the billions of microelectronic sensors in the emerging and disruptive technology of the Internet of Things (IoT). However, how the wide range of different types of hole extraction layers (HELs) impacts the indoor light harvesting of perovskite solar cells is still elusive, which hinders the material selection and industrial‐scale fabrication of indoor perovskite photovoltaics. In the present study, new insights are provided regarding the judicial selection of HELs at the buried interface of halide perovskite indoor photovoltaics. This study unravels the detrimental and severe light‐soaking effect of metal oxide transport layer‐based PV devices under the indoor lighting effect for the first time, which then necessitates the interface passivation/engineering for their reliant performance. This is not a stringent criterion under 1 sun illumination. By systematically investigating the charge carrier dynamics and sequence of measurements from dark, light‐soaked, interlayer‐passivated device, the bulk and interface defects are decoupled and reveal the gradual defect passivation from shallow to deep level traps. Thus, the present study puts forward a useful design strategy to overcome the deleterious effect of metal oxide HELs and employ them in halide perovskite indoor PVs.

## Graphical abstract

GA: Halide perovskite indoor photovoltaics have attracted enormous attention due to their application in the Internet of Things technology. This study investigates the indoor photovoltaic behavior of organic vs metal oxide hole extraction layers‐based halide perovskite devices, revealing the light soaking effect of metal oxide transport layers and the need for interface defect passivation to maximize indoor light harvesting.

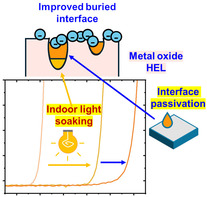

## 1 Introduction

**Figure 1:**
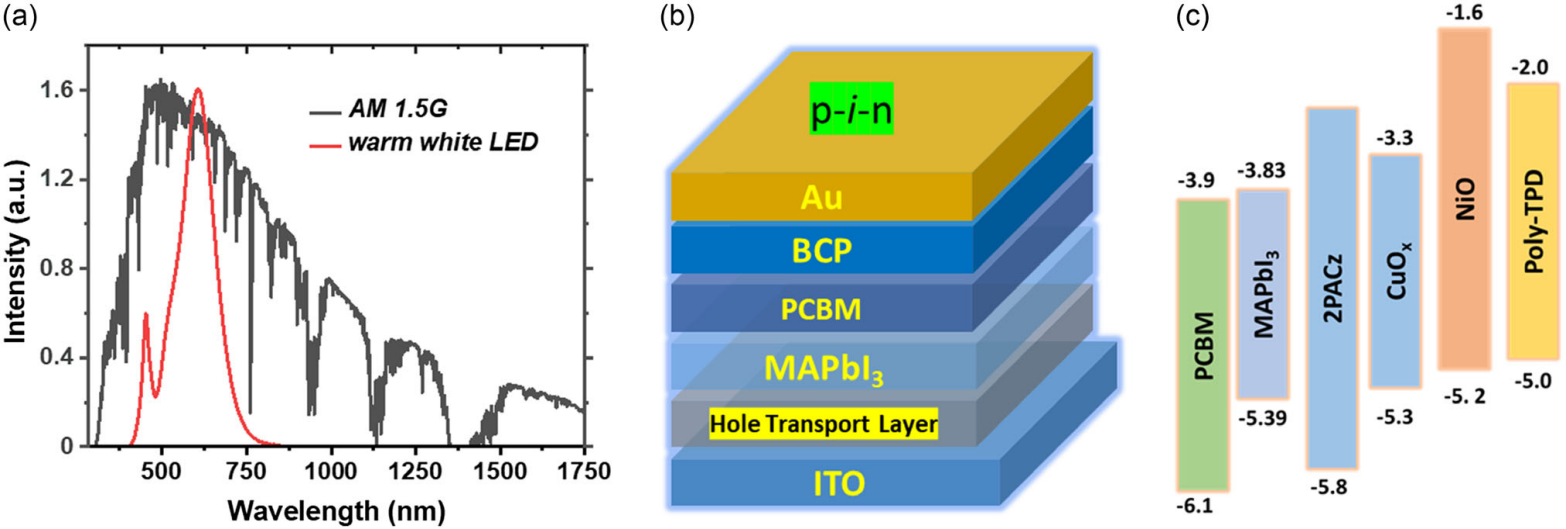
a) Comparison of the 1 sun spectrum with the warm white LED indoor light source (1000 lux) used in this study. b) Schematic of the investigated device structure. The hole transport layers utilized in this study are organic HEL (Poly‐TPD/PFN) and metal oxide HEL (NiO & CuO_*x*_). c) Schematic illustration of corresponding energy level diagram. The numbers shown are energy and the unit is ‘eV'.

With the explosive development of the Internet of Things (IoT) technology, indoor photovoltaics (IPV) are becoming a promising candidate to sustainably power the billions of wireless sensors for secured and smart buildings.^[^ 1, 2, 3, 4 ^]^ IPV refers to the devices that convert artificial light inside buildings to electricity. Indoor lighting in domestic and commercial buildings is mainly dominated by compact fluorescent lamps and white light‐emitting diodes (LEDs). Among the various photovoltaics (PV) technologies available today, halide perovskite‐based IPVs are most promising for integration with IoT technology because of their excellent optoelectronic properties, high specific power, earth‐abundance,^[^ ^5^ ^]^ easy, and cost‐effective processability using solution‐based methods such as roll‐to‐roll printing.^[^ ^6^ ^]^ Under standard 1 sun illumination, perovskite solar cells (PSCs) have made enormous progress within a decade with certified power conversion efficiency (PCE) exceeding 26%.^[^ ^7^ ^]^ This efficiency has been achieved by a focused research effort on optimizing the perovskite active layer, device architecture, and defect passivation methodologies across the bulk and buried interfaces. The main differences between IPV and the conventional 1 sun solar cells are in terms of illumination intensity and the light spectra, which are shown in **Figure** **1**a. The light intensity for indoor light (0.05–0.6 mW cm^−2^) is 500–1000 times lower compared to 1 sun (100 mW cm^−2^), leading to significantly different charge carrier dynamics and device design considerations. For example, the density of photo‐generated charge carriers is not high enough to fill the trap states that can exist in the bulk and at the buried interfaces.^[^ ^8^ ^]^ The absence of this beneficial light‐induced trap filling under indoor lighting demands stringent defect minimization approaches at every functional layer to maximize the PCE of IPVs. Efficient defect passivation will reduce the efficiency gap between the theoretically predicted (more than 55% depending on the indoor illumination source) and experimentally observed PCE of IPVs (≈35%, as mostly reported).^[^ 8, 9, 10, 11 ^]^ Most of the existing approaches to maximize the efficiency of IPV are focussed on halide perovskite active layer bandgap widening and interface passivation with less emphasis on the type of hole/electron transport layers and how it impacts the IPV performance.^[^ 8, 12, 13 ^]^ This is a very critical issue to address as recent studies have shown that the trap density at the buried interface can be two orders of magnitude higher than the bulk defect density and the charge transport efficiency depends on the quality of the charge transport layer/perovskite interface.^[^ 14, 15 ^]^

A typical PSC architecture consists of a perovskite light absorber sandwiched between a hole extraction layer (HEL) and an electron extraction layer (EEL). The n‐*i*‐p PSC with metal oxide (SnO_2_, TiO_2_) as EEL and 2,2′,7,7′‐tetrakis‐(N,N‐di‐*p*‐methoxyphenlamine)‐9,9′‐spirobifluorene (Spiro‐OMeTAD) as HEL has been the most successful architecture under 1 sun illumination. Under 1000 lux indoor LED light illumination, this architecture achieved the highest PCE of 41.2%. However, the n‐*i*‐p structure suffers from *J‐V* hysteresis and involves the complicated doping process for the HEL.^[^ 16, 17 ^]^ In this case, the emerging p‐*i*‐n planar structure is attracting more attention and becoming a promising device architecture to simplify the fabrication process and reduce the detrimental hysteresis effect.^[^ 18, 19 ^]^ Our previous study has shown that under indoor lighting conditions, p‐*i*‐n device architecture outperforms n‐*i*‐p architecture in terms of both efficiency and less hysteresis.^[^ ^17^ ^]^

In the p‐*i*‐n PSC devices, [6,6]‐Phenyl‐C61‐butyric acid methyl ester (PCBM) is the most commonly used EEL for its superior energy level matching and reduced *J‐V* hysteresis,^[^ ^20^ ^]^ and for extracting holes, poly(N,N′‐bis(4‐butylphenyl)‐N,N′bis(phenyl)benzidine) (poly‐TPD) and [2‐(9 H‐carbazol‐9‐yl)ethyl]phosphonic acid (2PACz) are the most promising and emerging organic HELs for high efficiency and simple fabrication.^[^ 21, 22, 23 ^]^ However, considering the resilience to oxygen and water ingression, metal oxide inorganic transport layers are promising candidates for efficient, stable, and cost‐effective p‐*i*‐n PSCs. Nickel oxide (NiO_*x*_) has attracted extensive investigation as the hole transport layer for its suitable bandgap, good energy level alignment, high stability, and high hole mobility.^[^ ^24^ ^]^ Under 1 sun, a PCE of 23.91% has been reported for NiO HEL‐based PSCs by Chen et al.^[^ ^25^ ^]^ Enhanced efficiency of above 25% has been achieved by employing passivated‐NiO to improve the buried interfacial conditions, indicating the superior potential of its hole extraction property under 1 sun irradiance.^[^ 26, 27 ^]^ Copper oxides (CuO_*x*_), which are also known as cupric oxide (CuO) and cuprous oxide (Cu_2_O),^[^ ^28^ ^]^ are alternative HELs for p‐*i*‐n photovoltaic systems with superior efficiency of 19% as achieved by Rao et al.^[^ ^29^ ^]^

Despite these promising attributes of organic and metal oxides as HEL under 1 sun, its performance comparison under indoor lighting conditions is still ambiguous. Moreover, insights on how the perovskite/HEL buried interface quality influences the photovoltaic PCE under different lighting conditions, its J‐V hysteresis properties, and light soaking effects are lacking in the context of halide perovskite IPVs. Such understanding is necessary for the development of reliable ambient light harvesters capable of self‐powering the IoT wireless sensors, which are currently absent in the literature and constitute the central theme of this study. In the present study, we investigate how the selection of HELs of organic (Poly‐TPD/PFN and 2PACz) and metal oxide (NiO & CuO_*x*_) based semiconductors in p‐*i*‐n perovskite IPV devices determines the light‐harvesting properties under different illumination levels. We observe that even though the photovoltaic properties of Poly‐TPD and NiO HELs‐based devices are comparable under 1 sun illumination, under indoor lighting, they demonstrate very different PV performance. We also observe that the devices employing a metal oxide transport layer suffer from lower performance, higher leakage current, and higher J‐V hysteresis compared to organic HEL‐based devices. The light soaking effect, which hasn't been previously reported for indoor perovskite PVs, was found to be necessary for metal oxide layer‐based devices to improve their performance. Since indoor light sources do not emit UV light and the light intensity is almost three orders lower compared to 1 sun irradiance, new insights have been obtained related to light‐induced defect passivation required for the halide perovskite IPVs. To alleviate the effect of traps, an interface modification strategy is applied by introducing a 2PACz‐based self‐assembled monolayer (SAM) resulting in higher PCE, better energetic alignment, much‐reduced leakage, and suppressed light soaking effect. Thus by systematically investigating the selection of the type of transport layers, charge carrier dynamics, and sequence of measurements from dark—light soaked—interlayer passivated device, we revealed the gradual defect passivation from shallow level to deep level traps. This is a new approach to analyzing bulk and interfacial defects by adjusting the light source and measurement conditions/sequence simultaneously, and it holds great implications for the halide perovskite‐based optoelectronic devices research community.

## 2 Results and Discussion

The PV devices were fabricated in a typical p‐*i*‐n device structure as shown in Figure 1b. All functional layers in the device were fabricated by solution‐processed spin‐coating method except the top metal electrode. Detailed experimental procedures of HEL preparation and device fabrication are in the Supporting Information. Figure 1c briefly illustrates the energy level diagram of different HELs and the perovskite photoactive layer of MAPbI_3_. The valence band/highest occupied molecular orbital (HOMO) level of the MAPbI_3_ and the HEL layers were measured using ambient photoemission spectroscopy (APS) as shown in Figure S1, Supporting Information. The measured values are in agreement with previous reports.^[^ 23, 28, 29, 30, 31, 32, 33, 34 ^]^

**Figure 2:**
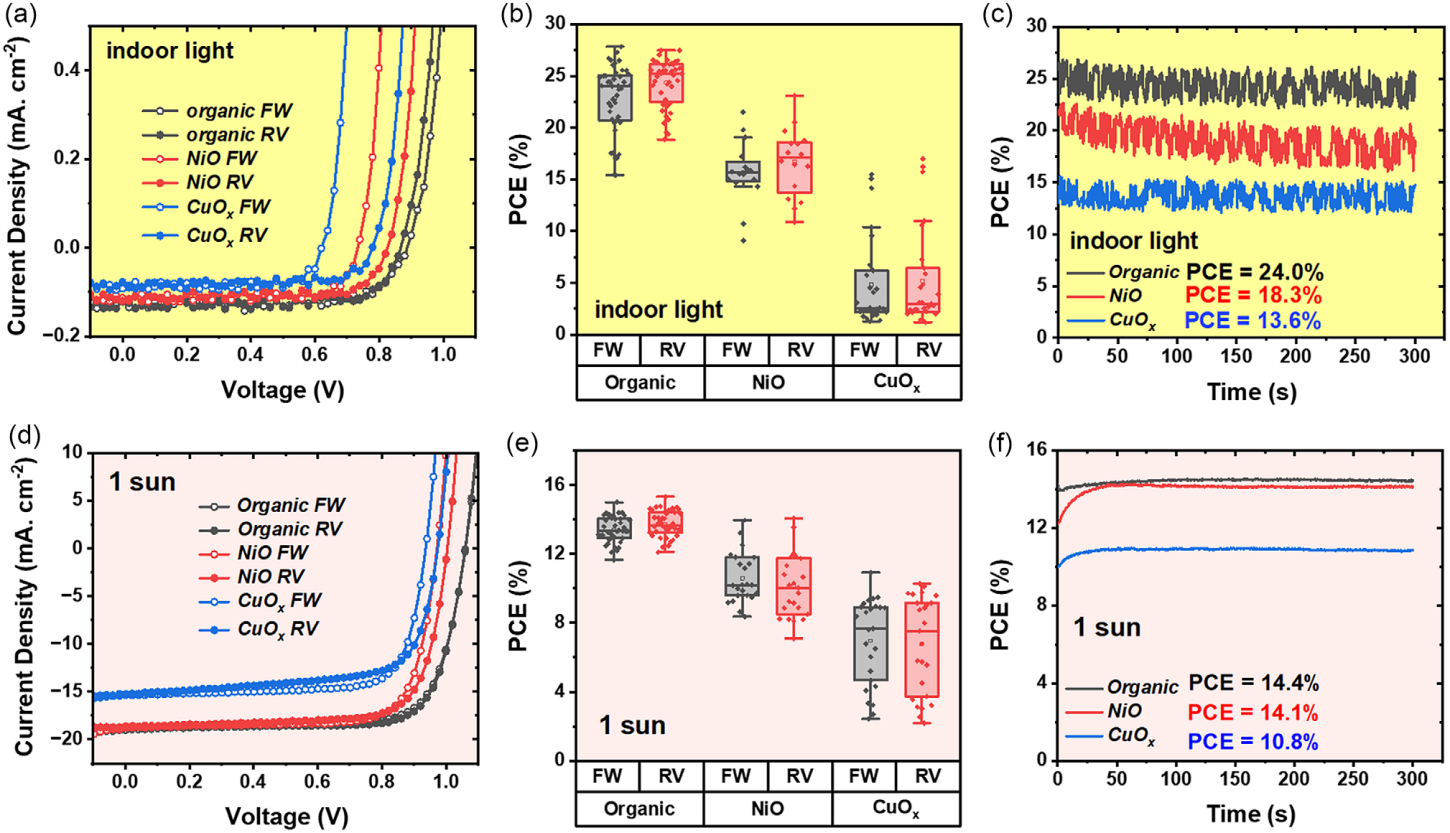
Results under indoor light illumination (top) and 1 sun illumination (bottom). a) Champion J‐V curves of different organic and metal oxide HELs‐based devices under indoor light illumination. b) The statistical distribution of PCE values of different HELs‐based devices. c) Steady‐state PCE comparison of different HELs‐based devices. d) Champion J‐V curves of different organic and metal oxide HELs‐based devices. e) The statistical distribution of PCE values of different HELs‐based devices. f) Steady‐state PCE comparison of different HELs‐based devices.

The *J‐V* characterization of the fabricated devices was carried out under both indoor and 1 Sun (AM 1.5 G) illumination conditions as shown in Figure 1a. All the indoor *J‐V* measurements were executed under 1000 lux, 2700 K warm white LED light (with irradiance 0.32 mW cm^−2^, noted as indoor light). The corresponding photovoltaic performance parameters are shown in **Figure** **2**, S2, Supporting Information, and **Table** **1**. For indoor measurements, photovoltaic devices comprising organic HEL exhibit an outstanding PCE of 27.84% and 26.58% for forward and reverse scans (Figure 2a), which is the highest among the three different HELs. NiO HEL device shows a champion indoor PCE of 21.50% and 23.10%. Compared to organic HEL‐based devices, NiO‐based devices exhibit slightly higher hysteresis, resulting from the discrepancy of *V*_OC_ (Figure S2, Supporting Information). The *V*_OC_ of the NiO champion device is 0.73 and 0.82 V for different scan directions, while the organic HEL device could reach 0.86 V for both forward and reverse scans. The drop in *V*_OC_ and the presence of *J‐V* hysteresis are severe for CuO_*x*_ HEL‐based devices (0.57 and 0.72 V for forward and reverse scans). A statistical distribution of PCE of organic and metal oxide HELs‐based devices is shown in Figure 2b. The consistently higher photovoltaic performance of organic HEL devices over the NiO and CuO_*x*_ HELs is evident from Figure 2b. CuO_*x*_ HEL‐based devices show a broad distribution of PCEs ranging from ≈2% to 15% with many devices showing PCE of ≈2%. By analyzing the distribution of photovoltaic performance parameters as shown in Figure S2a–c, Supporting Information, it can be noticed that the fill factor (FF) and short circuit current density (*J*_SC_) are comparable (only slightly lower for CuO_*x*_) for the three HELs, whereas the lower PCE values of the CuO_*x*_ HEL‐based devices are mainly due to the lower *V*_OC_ (which are only 0.1–0.2 V for many devices though some devices show 0.8 V). Figure 2c shows the steady‐state PCE measured from maximum power point tracking. The steady‐state PCE aligns well with the *J‐V* measurement PCEs of 24.0%, 18.3%, and 13.6% for PolyTPD‐PFN, NiO, and CuO_*x*_ HEL‐based devices. The good match between *J‐V* measurements and steady‐state PCE indicates the performance reliability of the fabricated PV devices. The high fluctuation in the indoor steady‐state PCE is due to the indoor light source being a domestic lamp rather than a standardized, voltage‐regulated light source like 1 sun solar simulator.

**Table 1:** Champion photovoltaic parameters from *J‐V* characterization of different HELs based devices.

		PCE [%]		FF [%]		*J*_sc_ [mA cm^−2^]		*V*_oc_ [V]	
		FW	RV	FW	RV	FW	RV	FW	RV
Indoor	Organic	27.84	26.58	77.49	75.27	0.133	0.131	0.86	0.86
	NiO	21.50	23.10	81.49	78.52	0.115	0.114	0.73	0.82
	CuO_*x*_	15.47	16.21	78.02	65.48	0.110	0.109	0.57	0.72
1 Sun	Organic	15.00	15.32	75.16	78.18	18.80	18.49	1.06	1.06
	NiO	13.95	14.01	76.04	75.10	18.86	18.66	0.97	1.00
	CuO_*x*_	10.91	10.26	75.61	69.06	15.41	15.25	0.94	0.97

In comparison, under 1 sun illumination, organic HEL‐based devices achieved a PCE of 15.0% and 15.32% under forward and reverse scans with negligible hysteresis, whereas NiO HEL devices showed a PCE of 13.95% and 14.01% (Figure 2d). However, CuO_*x*_ devices only showed PCEs of 10.91% and 10.26% with low *J*_SC_ of 15.41 and 15.25 mA cm^−2^, compared to ≈18 mA cm^−2^ for organic and NiO HEL‐based devices. Figure 2e shows the statistical distribution of PCE from these devices and the Poly‐TPD HEL‐based devices outperform the metal oxide HEL‐based devices. The CuO_*x*_ HEL devices show a larger spread in the PCE compared to the other two HELs (between 2% and 10% under 1 sun illumination). The statistical distribution of the performance parameters shown in Figure S2d–f, Supporting Information shows the main performance limiting parameters for the metal oxide HELs are *V*_OC_ and *J*_SC_. Figure 2f exhibits 1 sun steady state PCE of 14.4%, 14.1%, and 10.8% for organic, NiO, and CuO_*x*_ HEL‐based devices, respectively, which support the value from *J‐V* characterization. It took longer duration for NiO and CuO_*x*_ HEL‐based devices to reach stabilized PCE and this behavior can be attributed to the ion migration and charge trapping as has been previously reported.^[^ 17, 35 ^]^ Figure S3, Supporting Information shows the external quantum efficiency (EQE) spectra for the three groups of devices, and the organic HEL devices showed a higher EQE compared to metal oxide HEL‐based devices in general.

**Figure 3:**
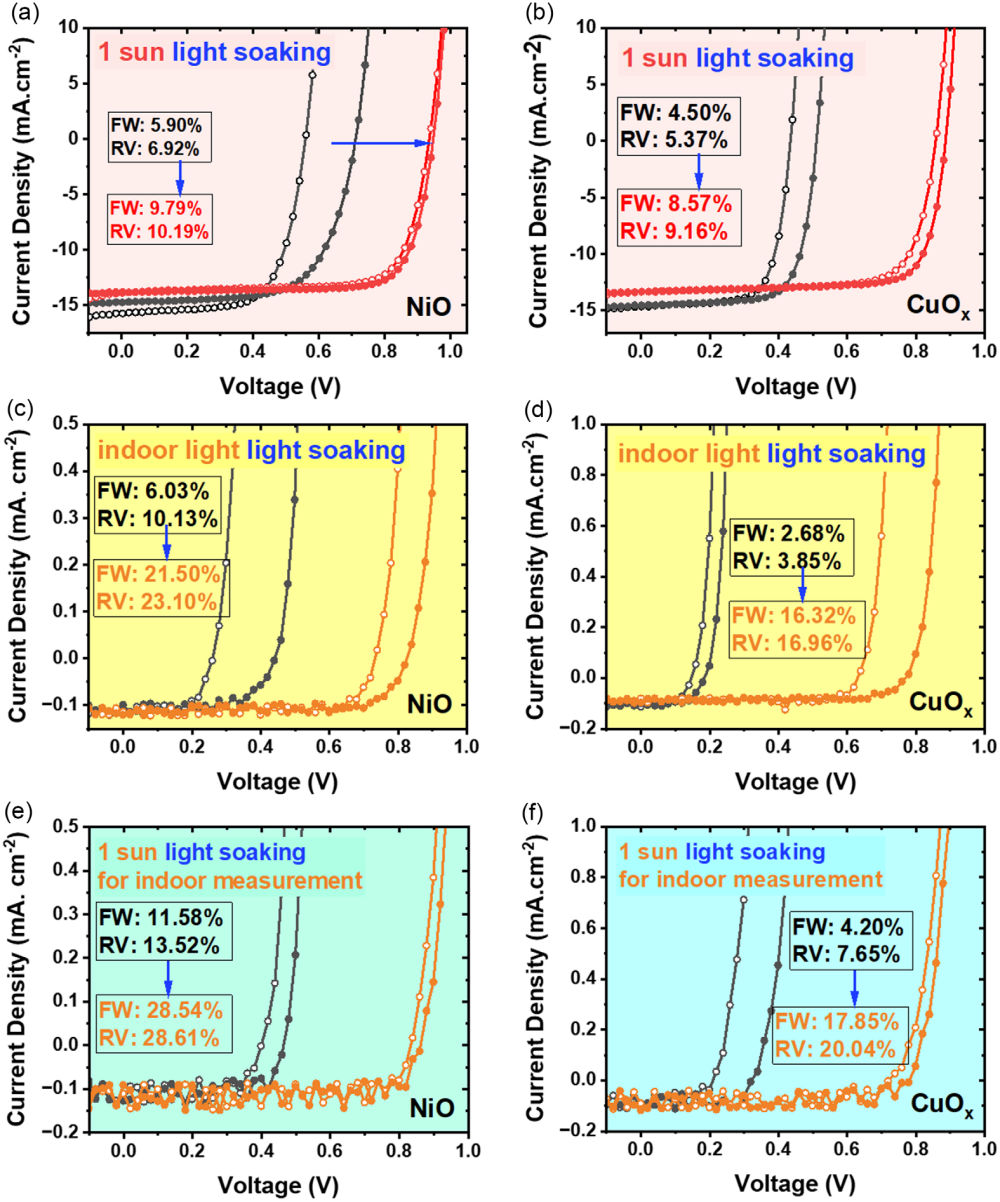
a) J‐V characteristics under 1 Sun illumination for the devices using NiO and b) CuO_*x*_ as HEL, respectively. c) J‐V characteristics under indoor illumination for the devices using NiO and d) CuO_*x*_ as HEL, respectively. e) Indoor J‐V characteristics after continuous sunlight illumination for the devices using NiO and f) CuO_*x*_ as HEL, respectively.

Next, the light soaking effect under 1 sun and indoor light, as shown in **Figure** **3**, was investigated. This effect refers to the evolution and improvement of photovoltaic performance parameters under light exposure.^[^ 36, 37 ^]^ Interestingly, the metal oxide HEL‐based devices do show light soaking effects, while no such effect was observed for the organic HEL‐based devices. From Figure 3a, under 1 sun exposure, NiO HEL‐based devices showed rather poor PCE of 5.90% (forward) and 6.92% (reverse) initially when the devices were just started to be illuminated from the dark. A significant improvement in PCE was observed after the devices were exposed to illumination for ≈5 min; the PCE increased to 9.79% and 10.19%, respectively. On the other hand, CuO_*x*_ devices also show an improved PCE from 4.50% and 5.37% to 8.57% and 9.16% for forward and reverse scans as shown in Figure 3b. From Figure 3a,b, during the evolution of PCE from the light soaking effect, the main photovoltaic parameter improved is *V*_OC_. For the NiO HEL‐based device, the *V*_OC_ was improved from 0.56 and 0.71to 0.94 and 0.95 V for forward and reverse scan, respectively [from Figure 3a].

Under indoor illumination, similar *J‐V* characteristics evolution to improved PCE is observed for the two metal‐oxide HEL‐based devices. The PCE of NiO HEL‐based devices increased from 6.03% FW (10.13% RV) to 21.50% and 23.10%, respectively (Figure 3c), whereas the PCE of CuO_*x*_ HEL devices increased from 2.68% and 3.85% to 16.32% and 16.96% (Figure 3d). No such light soaking effect has been observed for the organic HEL‐based devices even under indoor illumination. From Figure 3, it can be noticed that after light soaking, *J‐V* hysteresis is also suppressed for both NiO and CuO_*x*_ HEL‐based devices under both 1 sun and indoor light conditions. In both illumination conditions, the main photovoltaic parameters improved due to light soaking are *V*_OC_ (majorly) and FF (minorly) with no considerable effects in the *J*_SC_.

The light soaking effect has not been reported so far under indoor light illumination although it is commonly reported for PSCs under 1 sun illumination.^[^ 36, 38, 39 ^]^ The physical mechanisms responsible for the light soaking effect under 1 sun, as has been previously identified, are: 1) ion migration; 2) traps/defects at the bulk and buried interfaces; 3) charge accumulation at the electrode interface; and 4) lattice expansion (yet to be investigated in detail).^[^ ^38^ ^]^ Since the main performance parameters improved are *V*_OC_ and FF under both illumination conditions along with *J‐V* hysteresis suppression, the presence of ion migration coupled with the physical mechanisms of 2) and/or 3) might be the main contributing factor for the enhanced photovoltaic properties.^[^ ^38^ ^]^ The effect of halide ion migration from perovskite active layer to the HELs is also discussed previously.^[^ 40, 41, 42 ^]^ The *V*_OC_ and FF enhancement due to light soaking effect under 1 sun illumination has been attributed to the trap‐filling effect of photogenerated charge carriers both at the electrode interface and at the bulk and the increased built‐in field arising from the suppression of ion accumulations or the band bending effect at the interface. For indoor artificial lightsources, the intensity is almost three orders lowercompared to 1 sun illumination and the UV light is absent. Hence to understandthe main cause and mitigation of light soaking effects, the decoupling of bulkvs electrode interface‐related defects is necessary.

Previously, in organic PV and in PSCs consisting of metal oxide transport layers, such as TiO_2_, ZnO, etc, the presence of UV light in the sunlight has been crucial in observing the light soaking induced PV performance enhancement since the UV light with high energy photons could fill the deep level defects at the buried interface and transport layer.^[^ 36, 43, 44, 45 ^]^ Here in the present study, NiO is a wide bandgap (≈3.7 eV) p‐type metal oxide semiconductor. To decouple the contribution of perovskite/HEL interface defects from the bulk defects in the perovskite active layer, *J‐V* measurements were done in the following sequence. First, the devices were measured under indoor lighting conditions after storing in the dark, then the devices were rapidly moved under sunlight illumination for a period of 5 min. Finally, the devices were moved back to indoor light illumination and carried out the *J‐V* measurement immediately. In this way, we can get two indoor photovoltaic *J‐V* curves: *J‐V* curves directly measured without any light soaking; and indoor *J‐V* curves after continuous illumination with sunlight. If the photovoltaic parameters measured after sunlight illumination are improved just like the case for solely indoor continuous illumination, it demonstrates two things 1) that the low‐intensity indoor light sources can also alleviate light soaking effects; and 2) the defects in the HEL layer can also have a contribution in the light soaking effects. Figure 3e shows that the indoor PCE improved from 11.58% and 13.52% to 28.54% and 28.61% for NiO HEL devices after solely sunlight soaking. CuO_*x*_ HEL devices also show a significant improvement in indoor PCE from 4.20% and 7.68% to 17.85% and 20.04% as shown in Figure 3f. This consistent behavior confirmed that indoor light also plays a role in the light soaking effect to fill the bulk and interfacial traps. The light soaking under 1 sun further improves the performance of halide perovskite IPVs benefiting from the deep trap‐filling effect of UV light. Based on the previous studies, the bulk traps are shallow and mostly related to the perovskite active layer including at the buried interface, whereas the metal oxide interfacial transport layer‐related traps are deep.^[^ 36, 38, 39 ^]^ The observation of improved photovoltaic performance with light soaking under indoor lighting as well as 1 sun suggests the presence of both deep and shallow traps in the metal oxide HEL‐based photovoltaic devices.

**Figure 4:**
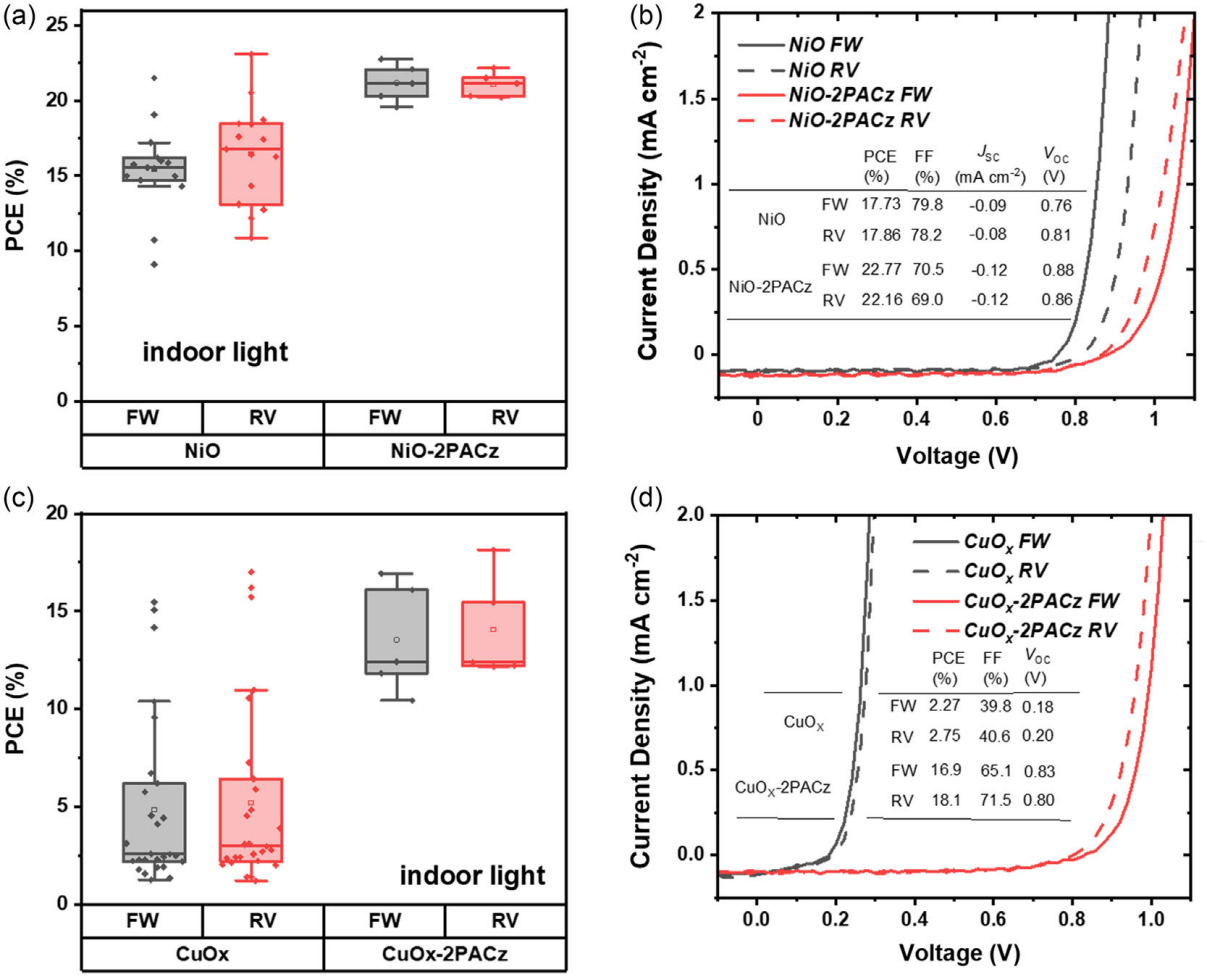
a) The statistical distribution of PCE values of devices with NiO HEL and 2PACz passivation layer under indoor light illumination. b) J‐V characteristics under indoor light illumination for the devices with NiO HEL and 2PACz passivation layer. c) The statistical distribution of PCE values of devices with CuO_*x*_ HEL and 2PACz passivation layer under indoor light illumination. d) J‐V characteristics under indoor light illumination for the devices with CuO_*x*_ HEL and 2PACz passivation layer.

To passivate these traps, an organic SAM approach was applied. The interfacial passivation effect of 2PACz ([2‐(9H‐carbazol‐9‐yl)ethyl]phosphonic acid) has been reported previously^[^ 23, 46, 47, 48 ^]^ and hence used in this study as an interlayer between metal oxide HEL and perovskite active layer. **Figure** **4**a,b shows that the average PCE for the NiO‐based devices enhanced after 2PACz modification. Typical *J‐V* characteristics present an improvement of PCE from 17.73% (forward) and 17.86% (reverse) to 21.77% (forward) and 22.16% (reverse) after interface modification. Figure S4, Supporting Information shows the distribution plots of photovoltaic performance parameters, revealing a significant enhancement of *V*_OC_ from 0.66 V for forward and 0.77 V for reverse scan to 0.86 for both scans after 2PACz modification. In addition, Figure S4b,e, Supporting Information indicates a slight improvement of *J*_SC_ and the uncompromised FF after modification with 2PACz. The same behavior is observed for the 2PACz‐modified CuO_*x*_ HEL‐based devices. Most of the low PCE (≈3%) devices for pristine CuO_*x*_‐based HEL exhibited enhanced PCE of above 10% after modification with 2PACz. From the *J‐V* curves shown in Figure 4d, it is obvious that the *V*_OC_ is the main photovoltaic performance parameter improved for CuO_*x*_/2PACz devices. The *V*_OC_ improved drastically from 0.18 and 0.20 to 0.83 and 0.80 V. The statistical distribution of photovoltaic performance of the corresponding devices (NiO and CuO_*x*_ HEL modified with 2PACz) under 1 sun illumination follows the same trend as under indoor illumination and this is shown in Figure S5, Supporting Information.

The photovoltaic devices with 2PACz as a stand‐alone HEL were also fabricated. Figure S6, Supporting Information shows the distribution of photovoltaic performance of the corresponding devices compared to that of poly‐TPD/PFN HEL‐based devices. The average PCE for 2PACz HEL obtained from Figure S6a, Supporting Information is 20.4% and 19.8%, respectively. The PCE for pristine 2PACz HEL is slightly lower compared to Poly‐TPD/PFN layer devices, and this might be due to the slightly deeper HOMO level of 2PACz, as shown in the energy level diagram in Figure 1c. It is important to note that the 2PACz passivated NiO devices show better performance (Figure 4a) compared to single 2PACz as HEL, showing its effectiveness as an interfacial modifier for the metal oxide transport layers. After modifying the metal oxide HELs with the 2PACz, no light soaking effects were observed for the corresponding photovoltaic devices, implying 2PACz passivated the interfacial and bulk defects.

**Figure 5:**
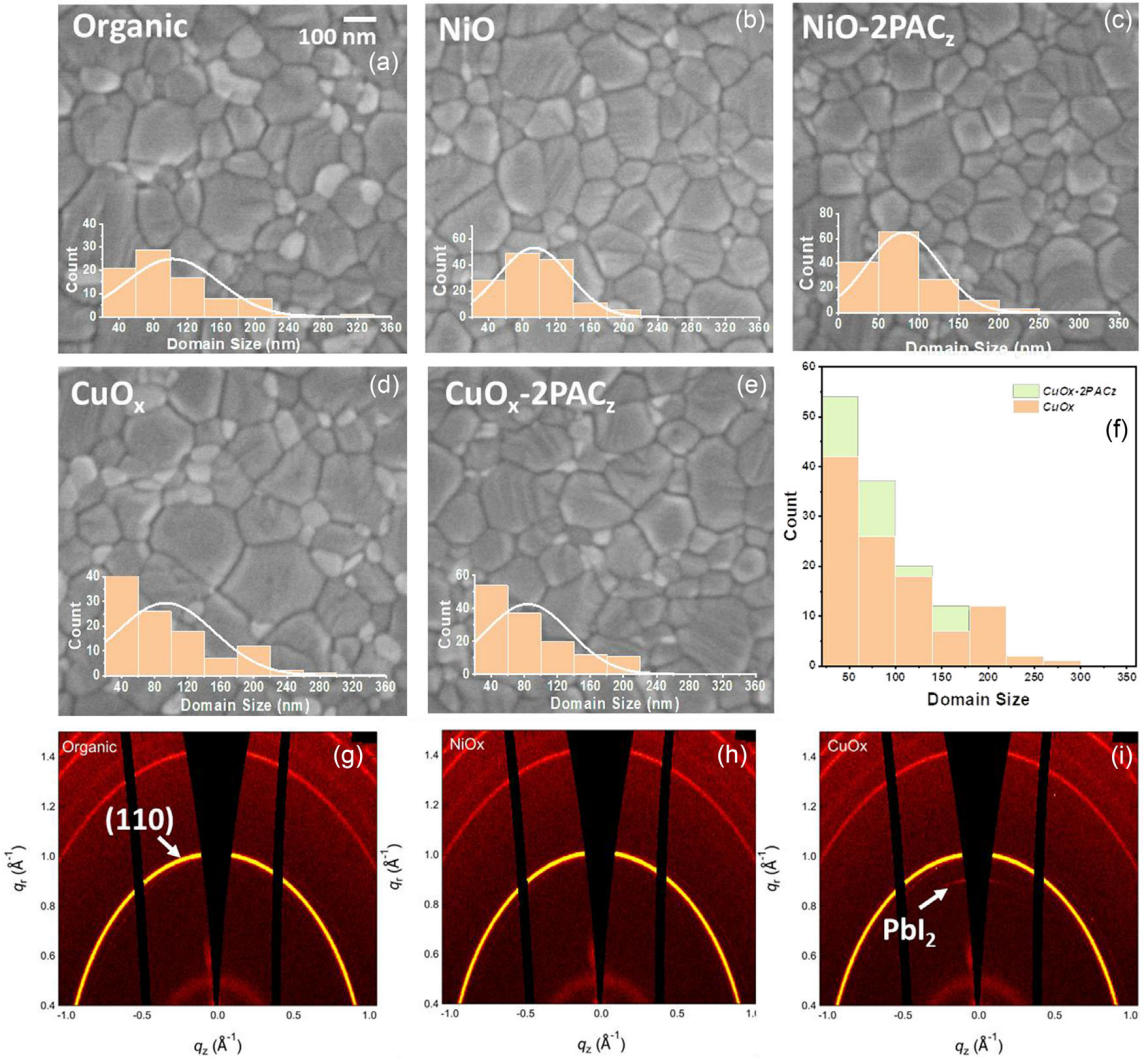
Scanning electron microscopy images of perovskite films on the top of a) organic transport layers, b) NiO, c) 2PACz passivated NiO, d) CuO_*x*_, and e) 2PACz passivated CuO_*x*_. f) Domain size histogram of CuO_*x*_ and 2PACz modified CuO_*x*_. GIWAXS data from perovskite layer grown on g) Poly‐TPD; h) NiO; i) CuO_*x*_.

To probe and understand any difference in properties of MAPbI_3_ grown on different HELs, detailed microstructural and optoelectronic characterizations were conducted on partial heterostructures and completed photovoltaic devices. **Figure** **5** shows the surface morphology of the MAPbI_3_ perovskite active layer grown on different transport layers. The films show similar grain‐like morphology but the domain size variation for MAPbI_3_ can still be observed from the histogram plots, which summarize the domain size for each case. This indicates that the perovskite crystallization is similar on organic, metal oxide, and passivated metal oxide layers, but the minor film morphology differences still exist in domain sizes and are worth paying attention to. Perovskite grains grown on organic transport layers exhibit rather concentrated domain size between 40 and 120 nm, which results in more uniform and dense films, while perovskite films on NiO and CuO_*x*_ layers exhibit scattered and inhomogeneous grain sizes in the range of 40–160 and 40–240 nm, respectively, as shown in the histograms from Figure 5a,b,d. After 2PACz modification of metal oxide transport layers, domain sizes are more consistent and homogeneous with a denser film morphology for MAPbI_3_ (Figure 5c,e). Figure 5f shows the domain size histogram of MAPbI_3_ perovskite grown on CuO_*x*_ and 2PACz passivated CuO_*x*_, respectively, and shows that the domain size for the latter is significantly more concentrated toward the lower size and the distribution is less broadened. The nonuniform domain distribution results from inhomogeneous nucleation and growth and can lead to the local crystal/grain misorientation causing nonradiative recombination losses.^[^ 49, 50 ^]^ The relatively higher surface energy values of metal oxide transport layers can promote heterogeneous nucleation of MAPbI_3_ compared to the low surface energy and smooth surfaced organic‐based HELs.^[^ 33, 51 ^]^

To investigate the crystalline property of the MAPbI_3_ layer grown on different HELs, X‐ray diffraction (XRD) measurements were carried out. As shown in Figure S7, Supporting Information, perovskite films grown on the three transport layers with and without 2PACz exhibit the same characteristic peaks at 14.1°, 28.5°, and 30.9°, corresponding to the (110), (220), and (222) planes of the tetragonal perovskite phase. This indicates that the interlayer does not modify the perovskite crystalline properties in general. In addition, 2‐dimensional grazing incidence wide‐angle X‐Ray scattering (GIWAXS), a critical technique to understand the structure‐property relationships, is performed. Compared to conventional XRD characterization, GIWAXS is more suitable for thin films as it can provide crystallographic information from both out‐of‐plane and in‐plane directions.^[^ ^52^ ^]^ The GIWAXS results for MAPbI_3_ perovskite thin films grown on different transport layer materials are shown in Figure 5g–i. Additional information from GIWAXS measurements is a PbI_2_ peak observed from the CuO_*x*_ sample, which was not detected in the XRD measurements. This could be due to the very weak PbI_2_ peak intensity, and hence lab‐based XRD technique may not be able to detect it. The excess PbI_2_ is usually located at grain boundaries and interfaces due to stoichiometry imbalance or perovskite degradation and can contribute to the poor performance of CuO_*x*_ HEL‐based devices as defect sites and charge transfer barriers.^[^ 16, 53 ^]^

**Figure 6:**
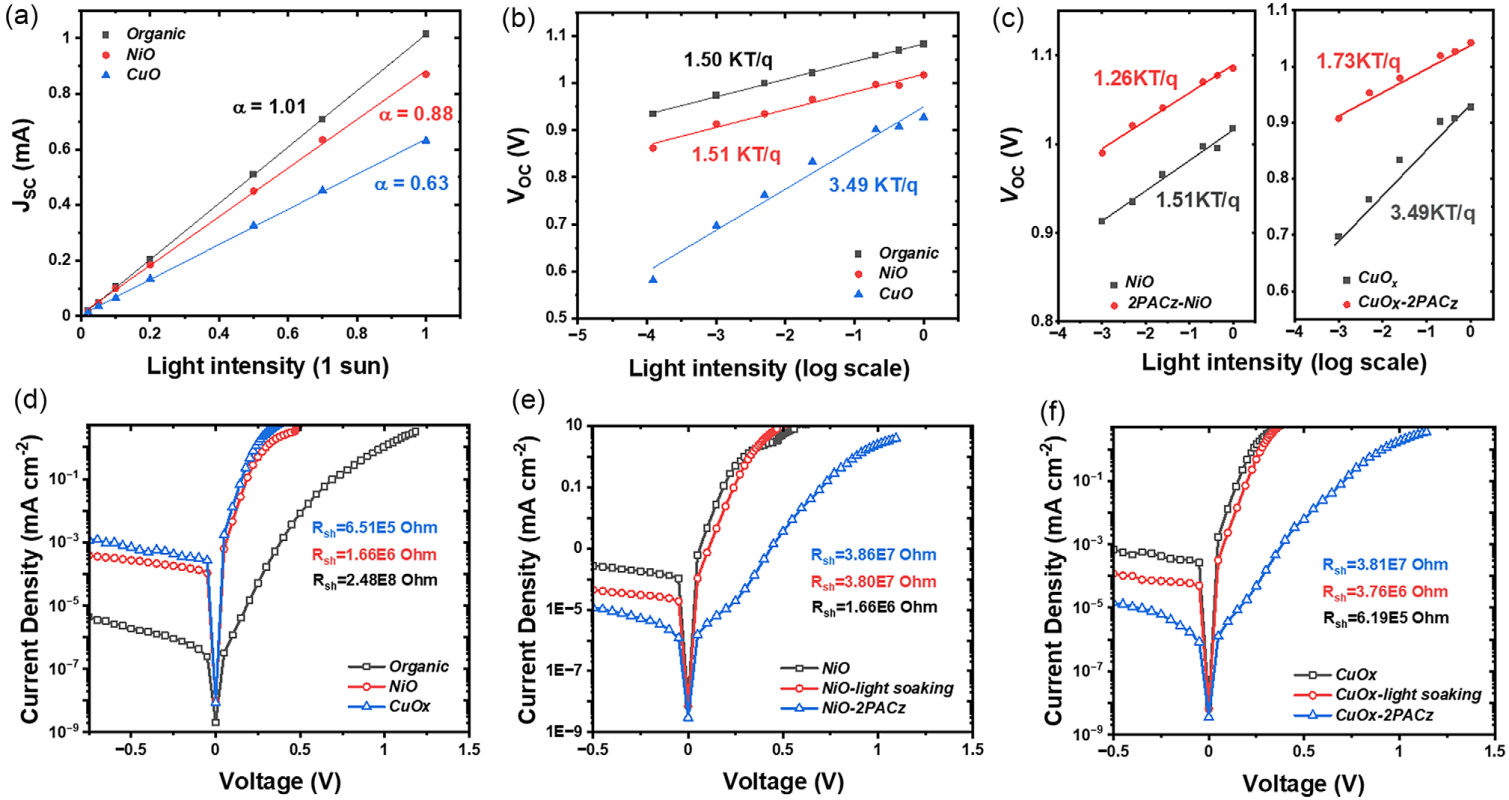
a) *J*_SC_ variation versus light intensity for organic transport layers, NiO, and CuO_*x*_ devices. b) *V*_OC_ variation versus light intensity for organic transport layers, NiO, and CuO_*x*_ photovoltaic devices. c) *V*_OC_ variation versus light intensity for NiO, 2PACz passivated NiO, CuO_*x*_, and 2PACz passivated CuO_*x*_ photovoltaic devices. d) Dark current density‐voltage curves for organic transport layers, NiO, and CuO_*x*_ devices. e) Dark current density‐voltage curves for NiO, NiO devices after light soaking, and 2PACz passivated NiO devices. f) Dark current density‐voltage curves for CuO_*x*_, CuO_*x*_ devices after light soaking, and 2PACz passivated CuO_*x*_ devices.

To gain more insight into the charge carrier dynamics of the photovoltaic devices fabricated using different HELs, recombination processes, trap density, charge carrier lifetime, and mobility were investigated. Light intensity‐dependent *J‐V* characteristics were performed to reveal the presence of nonradiative recombination losses. From **Figure** **6**a, organic HEL devices show a recombination factor α value of 1.013 ± 0.003, while for NiO and CuO_*x*_ devices, the recombination factors are 0.875 ± 0.010 and 0.633 ± 0.005, respectively. This result is consistent with the low *J*_SC_ value obtained from *J‐V* characteristic for metal oxide HEL devices as shown in Figure S2b,e, Supporting Information. The low recombination factors for metal oxide HEL devices suggest the existence of a charge extraction barrier at interfaces. The presence of defects is further evaluated by the dependence of *V*_OC_ on light intensity from Figure 6b. Organic HEL devices show an ideality factor (*n*_id_) of 1.50, whereas for NiO HEL devices, the *n*_id_ value is 1.51, and CuO_*x*_ HEL devices exhibit 3.49. High ideality factor >2 has been previously reported and is mainly attributed to the nonlinear shunt dominance, presence of tail states, or due to nonuniform spatial distribution of Shockley–Read–Hall recombination centers.^[^ 54, 55, 56, 57 ^]^ Thus the study of *V*oc and *J*sc dependence on light intensity results indicate that PV devices with CuO_*x*_ metal oxide HELs have higher recombination losses. Figure 6c shows the light intensity dependent measurements for *V*_OC_ for NiO and CuO_*x*_ after modification with 2PACz. It turns out that both ideality factors are reduced after 2PACz passivation: 1.51 to 1.26 for NiO, and 3.49 to 1.73 for CuO_*x*_, which suggests that trap‐assisted recombination is significantly suppressed. This recombination reduction can have three origins: 1) the improved surface coverage of the HELs, as the thickness of the NiO and CuO_*x*_ is below ≈5 nm; 2) the defect passivation at the buried interface; and 3) interfacial energetic modification. The passivation effect with 2PACz is more significant for CuO_*x*_ devices since the ideality factor is reduced for a factor of 2, which is consistent with *J‐V* characteristics results showing that the low PCE around 2% is boosted to 12.5% after introducing the 2PACz interlayer.

To understand the leakage current properties, dark current measurements and the comparison of the shunt resistance were carried out for the photovoltaic devices comprising different HELs. Figure 6d shows that the leakage current for PolyTPD/PFN HEL devices is 2.02 × 10^−9^ mA cm^−2^, which is the lowest among the three and with the highest shunt resistance of 2.48 × 10^8^ Ω cm^−2^. On the contrary, the high leakage current of 9.30 × 10^−9^ and 8.58 × 10^−9^ mA cm^−2^, and low shunt resistance of 1.66 × 10^6^ and 6.51 × 10^5^ Ω cm^−2^ for NiO and CuO_*x*_, respectively, were obtained for metal oxide HEL‐based devices. This is particularly detrimental for indoor photovoltaic applications since the shunt resistance effect dominates under dim light conditions. Figure 6e shows the evolution of dark current for pristine NiO HEL devices, NiO devices soaked under 1 sunlight, and 2PACz passivated NiO HEL devices. It reveals that the leakage current is the highest for the pristine NiO HEL‐based devices and is reduced after light soaking (from 9.30 × 10^−9^ to 7.20 × 10^−9^ mA cm^−2^), which can be due to the photogenerated charge carrier passivation of defects. After introducing 2PACz interlayer, the lowest leakage current (2.98 × 10^−9^ mA cm^−2^) is obtained. This reduced leakage current can be a result of interface defects passivation, improved built‐in voltage, and morphological homogeneity.^[^ ^58^ ^]^ The corresponding shunt resistance enhanced from single NiO HEL (1.66 × 10^6^ Ω cm^−2^) to light soaked device (3.80 × 10^7^ Ω cm^−2^), and finally to the 2PACz interlayer passivated device (3.86 × 10^7^ Ω cm^−2^). The same trend is also observed for CuO_*x*_ HEL devices as shown in Figure 6f. The leakage currents are reduced from 8.58 × 10^−9^ to 6.52 × 10^−9^ mA cm^−2^ with light soaking, and are further reduced to 3.53 × 10^−9^ mA cm^−2^ by 2PACz interlayer modification. CuO_*x*_ HEL devices exhibit low shunt resistance of 6.19 × 10^5^ Ω cm^−2^, which is enhanced to 3.81 × 10^7^ Ω cm^−2^ after 2PACz passivation.

**Figure 7:**
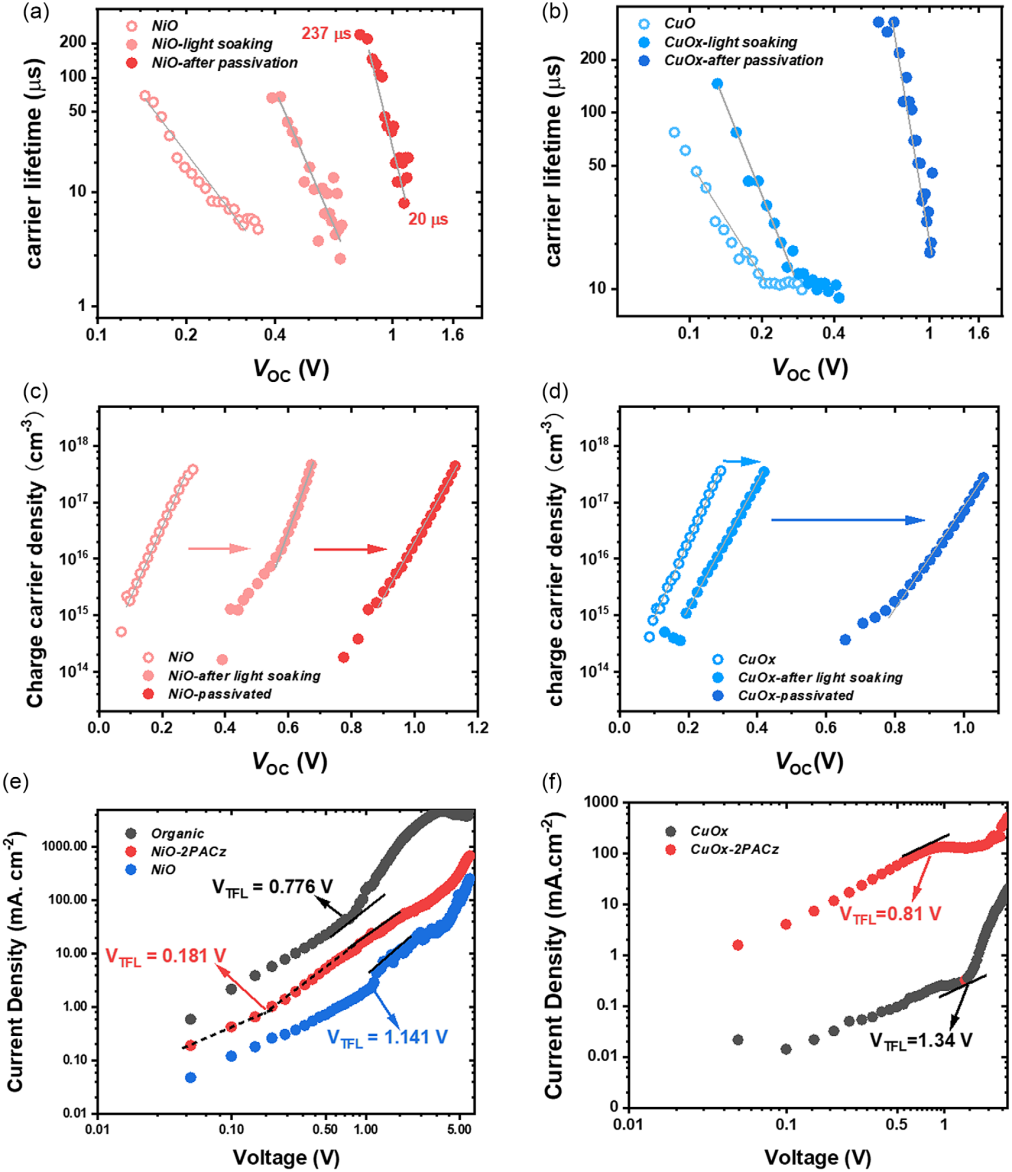
a) TPV characterization of NiO HEL devices, NiO devices after light soaking, and 2PACz passivated NiO devices. b) TPV characterization of CuO_*x*_ HEL devices, CuO_*x*_ devices after light soaking, and 2PACz passivated CuO_*x*_ devices. c) TPC characterization of NiO HEL devices, NiO devices after light soaking, and 2PACz passivated NiO devices. d) TPC characterization of CuO_*x*_ HEL devices, CuO_*x*_ devices after light soaking, and 2PACz passivated CuO_*x*_ devices. e) Space‐charge limit current model from organic, NiO, and 2PACz passivated NiO devices. f) Space‐charge limit current model from CuO_*x*_ and 2PACz passivated CuO_*x*_ devices.

To investigate the photogenerated charge carrier dynamics, transient photocurrent (TPC) and transient photovoltage (TPV) measurements were carried out. Both TPC and TPV measurements for NiO and CuO_*x*_ HEL devices were performed following the procedure of light soaking and interlayer modification. It is noticed from **Figure** **7**a that for NiO HEL‐only device, the *V*_OC_ is limited to a low range of 0.14–0.37 V, which is consistent with the initial *V*_OC_ value obtained from *J‐V* characteristics from Figure 3a,b; the *V*_OC_ value is increased to 0.7 V with higher carrier lifetime after light soaking, and then improved to above 1 V after 2PACz passivation. The 2PACz‐modified NiO HEL devices achieved a charge carrier lifetime of 237 μs at the irradiance level of 0.1 mW cm^−2^ (corresponding to the 0.7 V *V*_OC_ as shown in Figure 7a). This observation is well aligned with the corresponding *J‐V* characteristics consolidating the significance and effectiveness of interlayer passivation. Figure 7b shows the same behavior for CuO_*x*_ HEL devices where the *V*_OC_ is limited to 0.2 V for the pristine CuO_*x*_, with a lower charge carrier lifetime compared to NiO HEL device. However, after light soaking, the charge carrier lifetime is improved to 150 μs at maximum with an improved *V*_OC_ of ≈0.35 V. The CuO_*x*_ devices with 2PACz modification exhibit trap passivated *V*_OC_ close to 1 V. Figure 7c,d shows the TPC charge extraction results and we can observe that for every certain extracted charge carrier density, the *V*_OC_ from pristine metal oxide HEL‐based devices is the lowest, then improved after light soaking, and finally achieved a superior *V*_OC_ with 2PACz passivation. This indicates that for the same amount of photogenerated charge carriers, higher *V*_OC_ can be obtained by light soaking and with 2PACz interface modification.

To quantitively estimate the defect density in the perovskite films with different charge transport layers, space‐charge limited current (SCLC) measurements were performed. The HELs utilized are organic (Poly‐TPD/PFN), metal oxides, and 2PACz passivated metal oxides. A detailed description is given in the Supporting Information. Figure 7e,f shows the *J‐V* curves obtained from hole‐only devices of ITO/HEL/perovskite/P3HT/Au. The *J‐V* curve for pure 2PACz hole‐only device is shown in Figure S8, Supporting Information. The thickness of the perovskite active layer is 350 nm. Figure 7e shows that *V*_TFL_ of organic HEL device is 0.776 V, which corresponds to a calculated trap density of 1.79 × 10^16^ cm^−3^. NiO HEL devices exhibit a higher *V*_TFL_ of 1.14 V, which corresponds to higher trap density of 2.63 × 10^16^ cm^−3^. After 2PACz passivation, the trap density for NiO devices is reduced to 4.17 × 10^15^ cm^−3^. Similarly, Figure 7f shows that the trap density for CuO_*x*_ devices is reduced from 3.08 × 10^16^ to 1.86 × 10^16^ cm^−3^. The trap‐filled voltage (*V*_TFL_), the corresponding calculated trap density, and the hole mobility for different hole‐only devices are summarized in **Table** **2**.

**Table 2:** Trap‐Filled voltage and calculated trap density obtained from SCLC model.

	*V*_TFL_ [V]	Trap density [cm^−3^]	Hole mobility [cm^2^ V^−1^ S^−1^]
Organic	0.776	1.79 × 10^16^	1.39
NiO	1.141	2.63 × 10^16^	0.060
NiO‐2PACz	0.181	4.17 × 10^15^	0.24
CuO_*x*_	1.34	3.08 × 10^16^	0.017
CuO_*x*_‐2PACz	0.81	1.86 × 10^16^	0.62
Pure 2PACz	1.121	2.58 × 10^16^	2.05

MAPbI_3_ grown on organic transport layers has the highest hole mobility, 1.39 cm^2^ V^−1^ s^−1^, whereas for the NiO and CuO_*x*_ HELs, these values are only 0.060 and 0.017 cm^2^ V^−1^ s^−1^, respectively. Noticeably, the MAPbI_3_ grown on pristine 2PACz achieved very high hole mobility at 2.05 cm^2^ V^−1^ s^−1^, indicating the superior hole extraction property. The derived hole mobility could align well with the previous reports.^[^ ^59^ ^]^ After 2PACz interlayer modification, the hole mobility of MAPbI_3_ grown on NiO is improved from 0.060 to 0.24 cm^2^ V^−1^ s^−1^, whereas for CuO_*x*_‐2PACz, the hole mobility is improved from 0.017 to 0.62 cm^2^ V^−1^ s^−1^. The obtained result of improved charge carrier mobility after the front HTL/perovskite interface passivation agrees with the previous study by Alosaimi et al where the authors observed an increase in charge carrier transport properties after enhancing the quality of the ETL/perovskite interface.^[^ ^60^ ^]^

**Figure 8:**
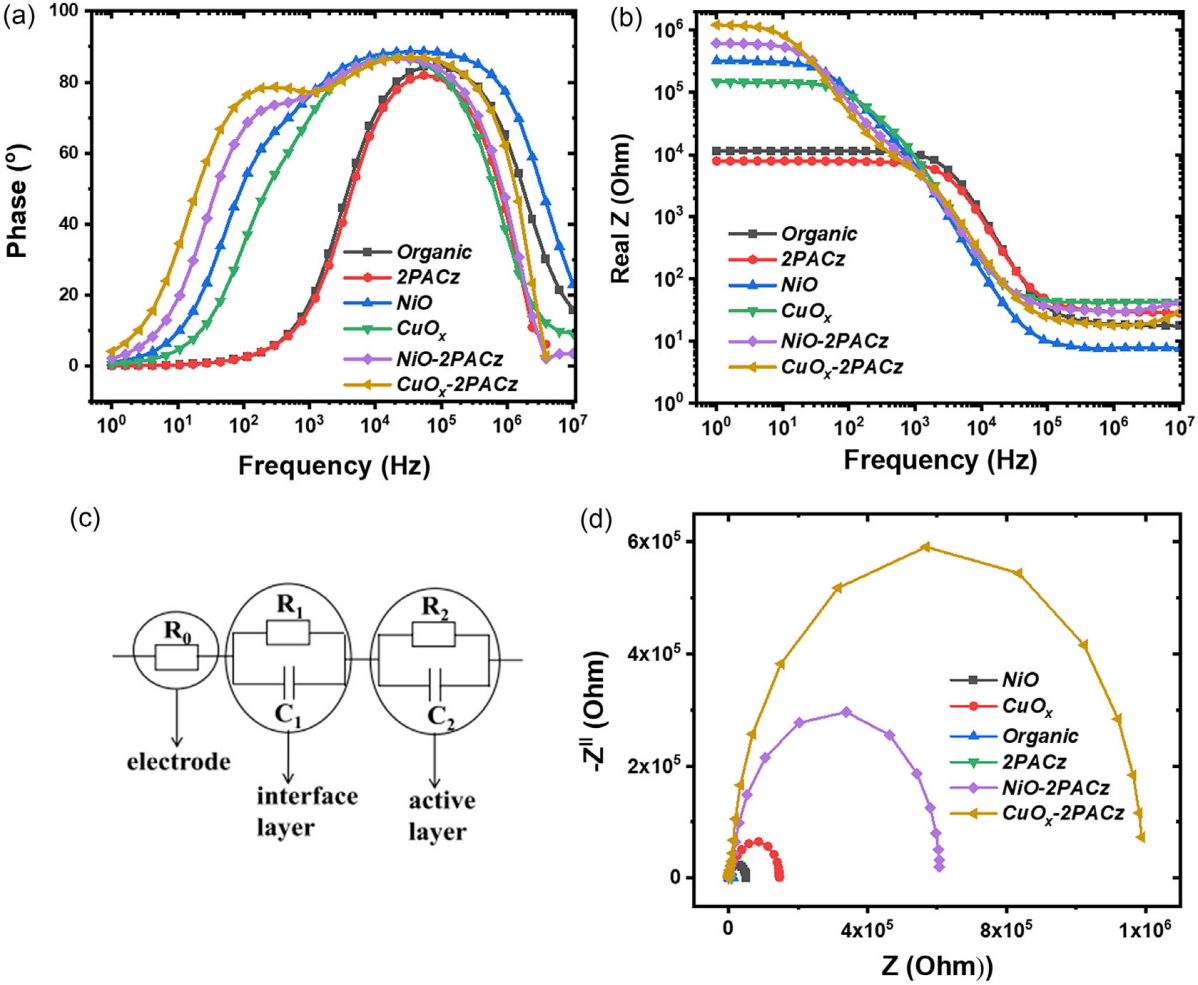
a) Phase vs. frequency Bode plots of the p‐*i*‐n PSCs with different front interfacial layers from EIS. b) Real impedance of the devices as a function of frequency from EIS. c) EIS equivalent circuit modeling. d) Nyquist plots of the p‐*i*‐n PSCs with different front interfacial layers from EIS.

The photoemission yield (APS) and work function (WF) measurements of the metal oxide HELs after 2PACz modification were carried out to understand the interface energetic alignment. As shown in Figure S9, Supporting Information, the WF of both metal oxides became more energetically favorable for hole transfer from the MAPbI_3_. Also, the deeper ionization potential of the 2PACz along with its molecular dipole moment can cause local p‐doping at the HTL/perovskite interface and beneficial band bending, which in turn can reduce the interface recombination losses.^[^ ^61^ ^]^ Overall, the 2PACz‐passivated NiO has better energy alignment compared to passivated CuO_*x*_ devices. To shed further insights on the bulk transport and interfacial resistance properties, electrochemical impedance spectroscopy (EIS) was performed on the fabricated PSCs based on different interlayers. The detailed description is given in the Supporting Information. The phase vs. frequency Bode plots of the p‐*i*‐n PSCs with different HELs are shown in **Figure** **8**a. In the case of devices with organic‐only HELs, such as in Poly‐TPD/PFN and 2PACz, mainly one relaxation process in the high‐frequency range (10^4^ Hz) is observed. Whereas in the case of devices with NiO or CuO_*x*_ as charge transport layers, the two peaks are evident: one at the high‐frequency range of 10^4^ Hz and the other at a relatively lower frequency of ≈10^2^ Hz, indicating the presence of two relaxation processes.^[^ 62, 63, 64 ^]^ When these oxide layers are covered with 2PACz transport layers, the two relaxation processes still exist, and their frequency range is also very similar to that of NiO/CuO_*x*_ HEL‐only devices. For the EIS carried out under dark conditions, the high‐frequency processes are related to the bulk transport of the perovskite film, and the low‐frequency processes are related to the perovskite/interface contacts (such as traps, ion migration, and accumulation).^[^ ^64^ ^]^ **Table** **3** lists the parameters obtained from EIS measurements.

**Table 3:** Parameters from electrochemical  impedance spectroscopy measurements.

Sample	Rs [Ohm]	R2 [k Ohm]	C2 [nF]	R1 [Ohm]	C1 [nF]	*Τ* (from phase plot) (HF/LF)
Poly‐TPD/PFN	18.1	11.4	4.29	–	–	1.98 μs
2PACz	29.6	7.8	5.8	–	–	2.75 μs
NiO	7.8	23.4	9	293 k	11.2	4.45 μs/1.3 ms
CuO_*x*_	42.8	22.8	11.1	122 k	8.72	8.60 μs/0.89 ms
NiO‐2PACz	33.8	14.3	8.85	594 k	12.6	7.0 μs/0.76 ms
CuO_*x*_‐2PACz	20.6	10.2	8.28	1.18	13.3	4.68 μs/0.76 ms

The corresponding real impedance of the devices as a function of frequency is given in Figure 8b. As seen in Figure 8b, the photovoltaic devices with only organic HELs such as Poly‐TPD and 2PACz show much lower impedance compared to devices with oxide only and metal oxide + 2PACz as HELs. Since in all these devices the EEL/perovskite interface is the same (MAPbI_3_/PC_60_BM/Ag), the differences in real Z with frequency (Figure 8b) are attributed to HEL/perovskite interface. To extract further information on the characteristic resistance and associated capacitance, the equivalent circuit modeling is applied to the Nyquist plots of the corresponding devices as shown in Figure 8c,d, respectively. For the devices with organic HELs, the interface resistances were negligible as in agreement with Figure 8a (only high‐frequency process), and hence for these devices, only one semicircle fitting is applied to the corresponding Nyquist plots. Table 3 summarizes the parameters obtained from EIS.

The transport resistance (R2) of the bulk perovskite is found to be the lowest for the devices with organic transport layers (11.4 and 7.8 k Ohm) and the highest for the oxide HEL‐based devices (23.4 and 22.8 k Ohm). This is in agreement with the SCLC measurements discussed above, which showed lower mobility for the MAPbI_3_ grown on metal oxide HELs, and the surface morphology characterization in Figure 5, which showed inhomogeneous domain distribution for the perovskite grown on oxide layers. This high bulk transport resistance can also account for the lower photovoltaic performance of the corresponding devices. However, when the metal oxide transport layers are passivated with the 2PACz layer, the bulk resistance decreases compared to that of the pristine oxide HEL alone (14.3 and 10.2 k respectively), in agreement with the higher mobility values as shown in Table 2.

## 3 Conclusion

Our study revealed the detrimental effect of metal oxide HELs in halide perovskite IPVs and the superior hole extraction property of the organic semiconductor‐based HELs. The devices with metal oxide HELs suffered lower PCE, higher *J‐V* hysteresis, and severe light soaking effects. The devices with organic semiconductor‐based hole transport layers demonstrated superior light‐harvesting properties under both indoor illumination and 1 sun. Detailed microstructural and optoelectronic characterization revealed that MAPbI_3_ grown on the metal oxide HELs has a higher density of traps, higher leakage current, lower mobility, and nonuniform distribution of domains. However, the interface engineering using the 2PACz SAM eliminated the detrimental light soaking effect and dramatically enhanced indoor light harvesting by suppressing the leakage current and passivating the defects. Our study demonstrated a promising route to employ the stable metal oxide charge transport layers in halide perovskite IPVs by interface engineering and provides important device design strategies for the selection of appropriate charge extraction layers to maximize the indoor light harvesting.

## Conflict of Interest

The authors declare no conflict of interest.

## Supporting information

Supplementary Material

## Data Availability

The data that support the findings of this study are openly available in 2 at https://www.[url/doi], reference number 62. These data were derived from the following resources available in the public domain: [Resource 1], https://www.[resource1]; [Resource 2], https://www.[resource2]. The research data underpinning this publication can be accessed at https://doi.org/10.17630/3a553e5b-d66b-4952-a1f5-2869d17a2418.
