# Supplementary material for: Metal Oxide vs Organic Semiconductor Charge Extraction Layers for Halide Perovskite Indoor Photovoltaics
Source: Small Sci. 2024 Sep 10;4(12):2400292. doi: 10.1002/smsc.202400292 (PMC11935188; doi:10.1002/smsc.202400292)
Supplement: Supplementary file 1 — Supplementary Material [file SMSC-4-2400292-s001.pdf]

Supporting Information

Metal oxide *vs* Organic semiconductor Charge Extraction Layers for Halide Perovskite Indoor Photovoltaics

Shaoyang Wang[1], Tim Kodalle[2], Sam Miller[1], Carolin M. Sutter-Fella[2], and Lethy Krishnan Jagadamma[1*]

[1]Energy Harvesting Research Group, School of Physics & Astronomy, SUPA, University of St Andrews, North Haugh, St Andrews, Fife, KY16 9SS, United Kingdom

[2]Molecular Foundry, Lawrence Berkeley National Laboratory, California 94720, USA, United States

[3]Advanced Light Source, Lawrence Berkeley National Laboratory, 1 Cyclotron Road, Berkeley, California 94720, USA

*Email for correspondance: lkj2@st-andrews.ac.uk

Materials: CH_3NH_3I was purchased from Greatcell solar. PbI_2 was purchased from Tokyo Chemical Industry, $PbBr_2$ and $PbCl_2$ were purchased from Alfa Aesar. For the electron transport layers, bathocuproine (BCP) was acquired from Sigma Aldrich (99.99% purity), and $PC_{60}BM$ was from American Dye Source Inc. For the hole transport layers, poly(4-butyltriphenylamine) (poly-TPD) was aquired from American Dye Source Inc., poly[(9,9-bis(3'-(N,N-dimethylamino)propyl)-2,7-fluorene)-alt-2,7-(9,9-dioctylfluorene) (PFN) was bought from 1 Materials. 2PACz ([2-(9H-carbazol-9-yl)ethyl]phosphonic acid) was purchased from Tokyo Chemical Industry. Nickel oxide was purchased from Avantama AG. Copper (ii) acetylacetonate ($Cu(acac)_2$, purity >99.9%) was purchased from Sigma Aldrich. Solvents dimethyl sulfoxide (DMSO, anhydrous, ≥99.9%), N,N-dimethylformamide (DMF, anhydrous, 99.8%), chlorobenzene (anhydrous, 99.8%), chloroform (anhydrous, 99.8%), methanol (anhydrous, 99.8%), acetonitrile (anhydrous, 99.8%), and diethyl ether (anhydrous, ≥99.7%) were purchased from Sigma Aldrich.

Device fabrication: The $MAPbI_3$ precursor solution was prepared by dissolving 159 mg MAI and 461 mg PbI_2 into 66 μL DMSO and 636 μL DMF, stirring under room temperature for 1 h. Pre-patterned indium tin oxide (ITO) coated glass substrates were cleaned by sodium dodecyl sulphate (SDS), deionized water, acetone and isopropyl alcohol

with sonication sequentially. The substrates were then cleaned with UV Ozone cleaner for 20 min. For organic extraction layers, 1.4 mg Poly-TPD was dissolved into 1 mL and stirred continuously for 4 hours under room temperature, 100 μL Poly-TPD was spin-coated onto the cleaned ITO substrate at 6000 rpm for 30 s. 1 mg PFN-P1 was dissolved into 995 μL methanol, stirring for 2 h under 60 °C, then 5 μL acetic acid was added and stirring for another 2 h under 60 °C. 100 μL PFN was spin-coated on top of Poly-TPD at 3000 rpm for 30 s. For the metal oxide extraction layers, NiO suspension was prepared by diluting 2.5 wt% nanoparticle solution using ethanol at a ratio of 1:10. 2 mg $Cu(acac)_2$ was dissolved into 1 mL chloroform under room temperature for 3 h. CuO_x film was prepared by spin-coating 90 $c(acac)_2$ solution at 2000 rpm for 30 s, following 120 °C annealing for 20 min. The film was then washed with 2 mL anhydrous methanol and cleaned by UV Ozone cleaner for 20 min. For 2PACz solution, 2.75 mg 2PACz was dissolved into 1 mL methanol, stirring at 38 °C for 15 min. 100 μL 2PACz solution was spin-coated on top of metal oxide extraction layers at 3000 rpm for 30 s, followed by 100 °C thermal annealing for 10 min. The $MAPbI_3$ perovskite film was spin-coated on the top of PFN layer at 4000 rpm for 30 s, anti-solvent washing was carried out at 7 s after the spin coating started by using 750 μL diethyl ether. The spin-coated perovskite thin film was thermal annealed at 100 °C under vacuuming for 1 min and then annealed under N_2 for another 2 min. For hole extraction layers, 13 mg $PC_{60}BM$ was dissolved into 1 mL chlorobenzene, stirring at 60 °C for 4 h. The $PC_{60}BM$ solution was filtered with 0.2 μm filter before use. 100 μL $PC_{60}BM$ was spin-coated at 1000 rpm for 60 s, annealing at 80 °C for 5 min. BCP solution was prepared by dissolving 0.5 mg BCP into 1 mL anhydrous ethanol, stirring at room temperature for 4-5 h. 100 μL BCP was spin-coated on top of $PC_{60}BM$ at 4000 rpm for 30 s. Finally, 100 nm Ag electrode was deposited on the top by thermal evaportation.

Characterisation of the devices and films: For current-voltage characteristics, 1 Sun measurement was carried out using a solar simulator with Xenon Arc lamp (150 W, 50 × 50 mm, Class AAA, Sciencetech Solar simulator) at the light intensity of 100 mW/cm^2 (AM 1.5G). The indoor illumination measurements were carried out using a 'warm white' LED bulb with colour temperature 2700 K. The intensity of the warm white LED was fixed to 0.32 mW/cm^2, corresponding to 1000 lux. The irradiance level was checked using an Optometer P9710. The current-voltage characterisation was measured with Ossila Source Measure Unit (SMU) and an Ossila Solar Cell IV software. The devices were masked by a metal mask with an aperture of 0.05 cm^2 to define the active area of photovoltaic cell. The devices are measured under a bias scan of -0.1 V to 1.2 V range (forward scan), followed by

a reverse scan of 1.2 V and -0.1 V, with a voltage setting time of 0.2 s. The voltage increment of the *J-V* characteristic was 0.05 V and the scan rate was 0.2 V/s. For the light soaking measurements also the same biasing conditions are applied and the light soaking process was carried out for the respective photovoltaic devices under open circuit conditions along with the exposure of the respective light sources. For the steady state PCE measurement, the devices were characterized by applying a voltage bias equal to the voltage at maximum power point for 5 minutes. TPV and TPC measurements are carried out with the characterization platform, Paios, Fluxim AG, Switzerland. For TPV and TPC measurement, V_{OC} perturbation is induced by a transient light pulse of 500 μs, and the intensity of which is 10% of the light intensity. The settling time before the light pulse is applied and the follow-up time after the light pulse is 6 s and 1 ms, respectively.

SCLC measurement is carried out using Ossila Source Measure Unit (SMU) and an Ossila Solar IV software. The *J-V* curves from SCLC measurements show three distinct regions: the ohmic region, trap-filled limit region and Child's region[1]. A trap filled limit (TFL) can be identified at the transition point from ohmic region to trap-filled limit region. The trap-filled voltage (V_{TFL}) is related to the trap density (N_t) within the devices following the relation of[1],

$$V_{TFL} = \frac{eN_t d^2}{2\varepsilon\varepsilon_0}$$

where e is the elementary charge, d is the thickness of perovskite active layer, ε is the dielectric constant of the perovskite active layer which is 25.5, and ε_0 is the permittivity of free space[1].

In the Child's region of SCLC which is marked by the tangent in Figure 8 (e), the charge carrier mobility can be derived from Mott-Gurney law[2]:

$$J = \frac{9\varepsilon\varepsilon_0 \mu V^2}{8d^3}$$

where d is the thickness of perovskite active layer, ε is the dielectric constant of the perovskite active layer which is 25.5, and ε_0 is the permittivity of free space.

Light intensity dependent measurement was carried out with the same solar simulator by adjusting the aperture for light intensity variation. Transient photovoltage, transient photocurrent, space-charge-limited-current measurement, leakage current measurement and

electrochemical impedance measurement were carried out with a characterization platform, Paios, Fluxim AG, Switzerland.

The frequency range considered is 0.1 Hz to 10 MHz for the EIS measurements. The high frequency relaxation processes are attributed to the transport related effects and the low frequency relaxation processes are related to the buried interface related in the completed photovoltaic devices. The characteristic time scale of these relaxation processes at high frequency range were estimated [from the Figure 8 (a)] based on the following formula[3,4],

$$\tau = \frac{1}{2\pi f_p}$$

where f_p is the peak frequency.

The interface-related resistance is found to be higher for the oxide and 2PACz modified metal oxide transport layers compared to organic-only HELs. Even though the interface-related resistance is found to be higher for the 2PACz modified metal oxide layers, the improved performance compared to pristine oxide HELs can be related to the reduced transport resistance for such devices along with the interface transfer times (estimated from the phase plots as given in Table 3). This reduced transport resistance is in accordance with the improved homogeneous surface morphology observed for $MAPbI_3$ layers grown on 2PACz modified metal oxide HELs.

X-ray diffraction spectra of the perovskite thin films were collected on Bruker D8 Discover (EIGER2R-500K 2d detector) instrument using Cu Kα1 (λ=1.54060 Å). Data were collected in the range 5° −60° 2θ with a step size of 0.02° and a time step of 1 s, cumulative time per step (2d detector) of 623 s. The GIWAXS data were collected at the 12.3.2 microdiffraction beamline of the Advanced Light Source (ALS). The respective perovskite films were pre-prepared on patterned ITO-on-glass substrates coated with different HELs and placed on a completely adjustable sample stage. The incident angle of the incoming X-ray beam was set to 2° with an accuracy of about 0.1° and a beam energy of 10 keV was used. The sample detector distance (SDD) was ≈155 mm and the detector was positioned at an angle of 35° from the sample plane. The GIWAXS data were recorded using a Pilatus 1 M 2D detector (Dectris Ltd.) and an exposure time of 10s. The measured GIWAXS frames were calibrated using an Al_2O_3 reference sample. Data evaluation was done using software written in house.

Ionisation potential measurements were made using an SKP5050 Scanning Kelvin Probe with an APS04 bolt-on module to perform ambient photoemission spectroscopy. This device uses a deuterium lamp with a built-in monochromator which scans the sample using deep ultraviolet light between 3.4-7.0 eV. The cube root of the raw photocurrent data is then taken, in accordance with Fowler's theory of photoemission, before an extrapolation fit is performed on the linear region of the resulting data set. The intersection of this linear extrapolation with the baseline is the ionisation potential of the semiconductor being studied.

The scanning electron microscopy (SEM) images of the hybrid perovskite layers were taken using a Hitachi S4800 scanning electron microscope.

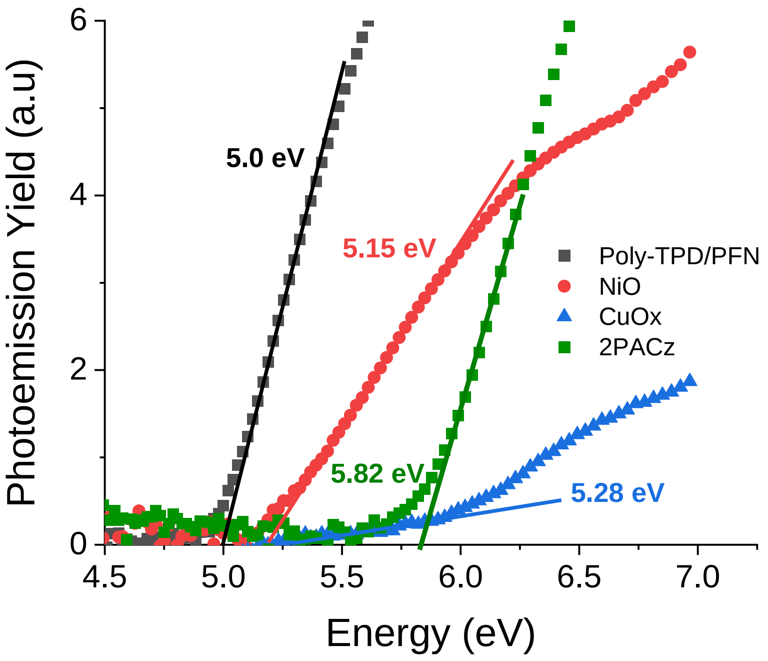

Figure S1. Ambient photoemission spectra of Poly-TPD/PFN, 2PACz, NiO and CuO_x thin films

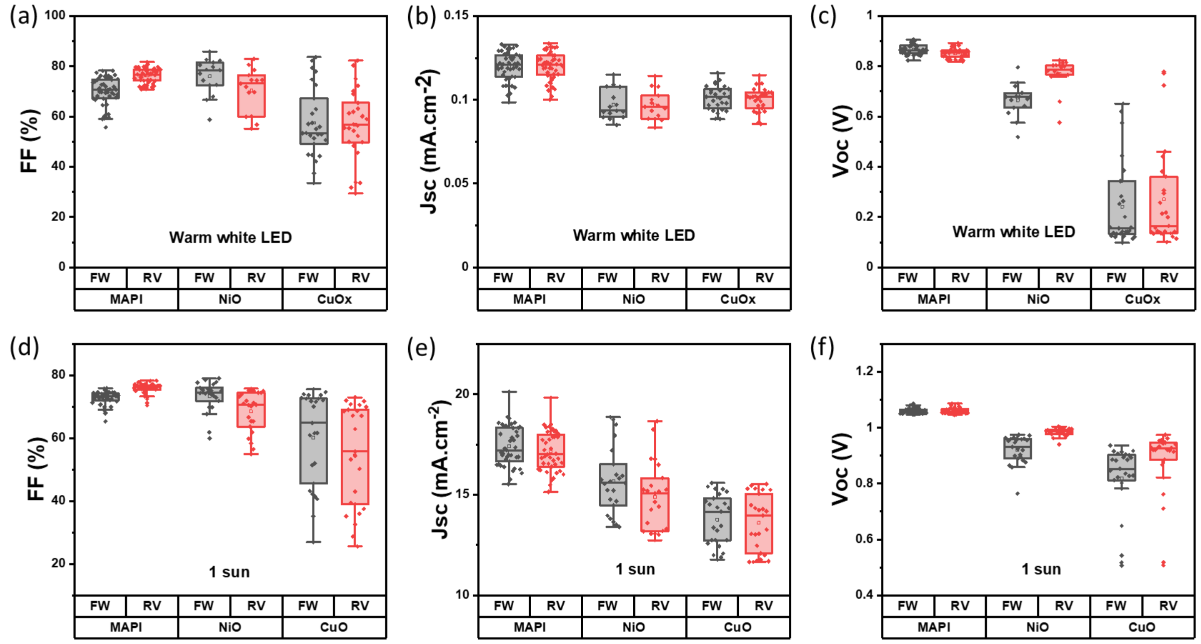

Figure S2. Indoor photovoltaic parameters of (a) fill factor, (b) J_{SC} (c) V_{OC} for organic, NiO and CuO_x HEL-based devices. 1 sun photovoltaic parameters of (d) fill factor, (e) J_{SC} (f) V_{OC} for organic, NiO and CuO_x HTL-based devices.

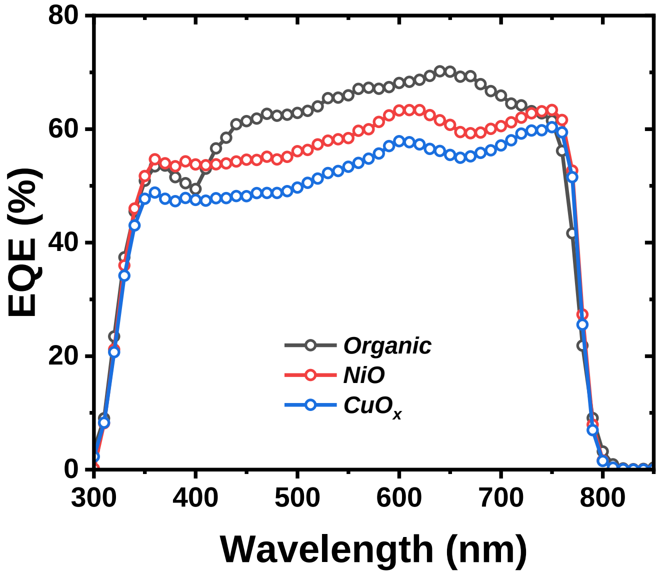

Figure S3. EQE spectra of organic (poly-TPD/PFN), NiO and CuO_x HELs-based devices.

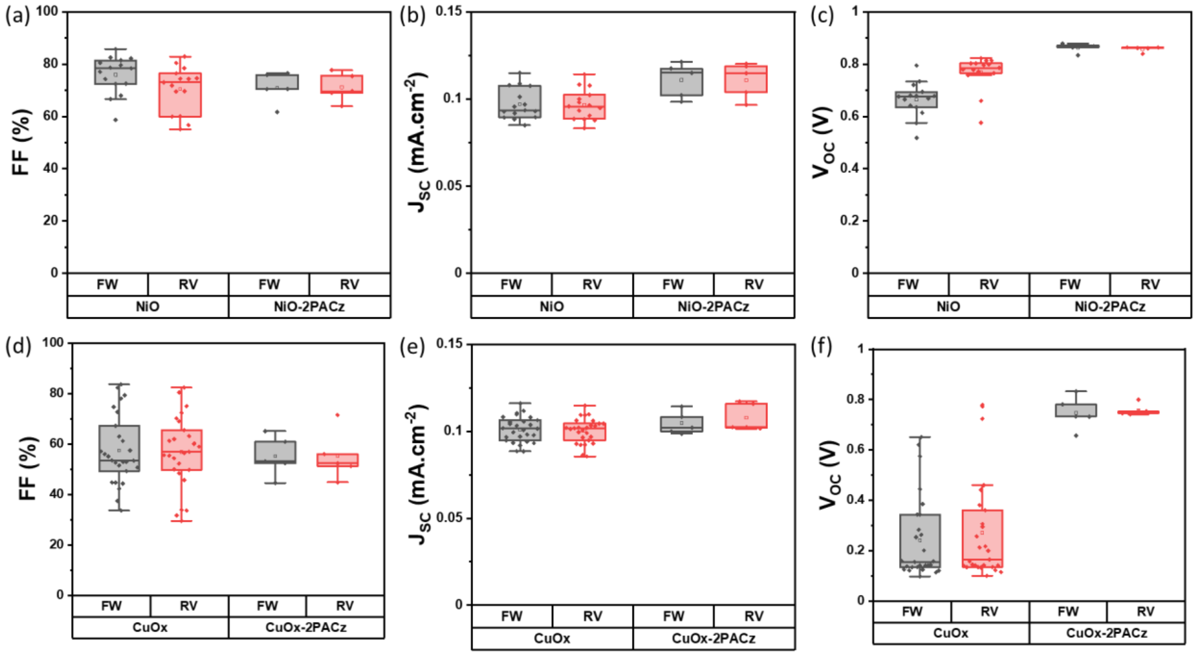

Figure S4. Indoor photovoltaic parameters of (a) fill factor, (b) J_{SC} (c) V_{OC} for NiO and 2PACz passivated NiO-HEL based devices. Indoor photovoltaic parameters of (d) fill factor, (e) J_{SC} (f) V_{OC} for CuO_x and 2PACz passivated CuO_x-HEL based devices.

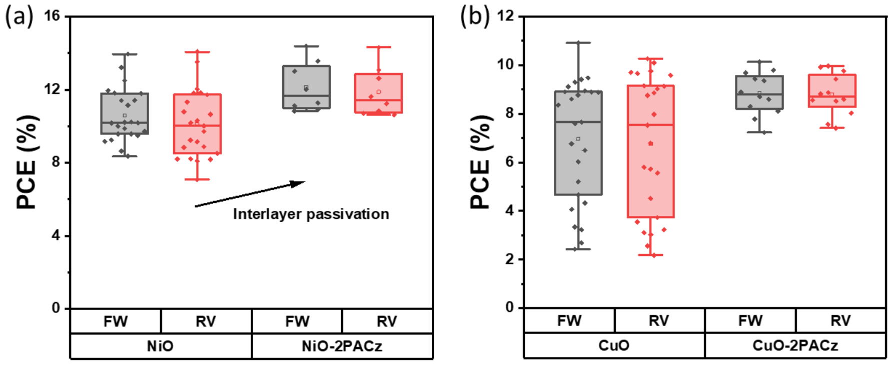

Figure S5. (a) The distribution plots of PCE under 1 sun illumination of pristine NiO HEL based devices and 2PACz modified NiO devices. (b) The distribution plots of PCE under 1 sun illumination of pristine CuO_x HEL devices and 2PACz modified CuO_x devices.

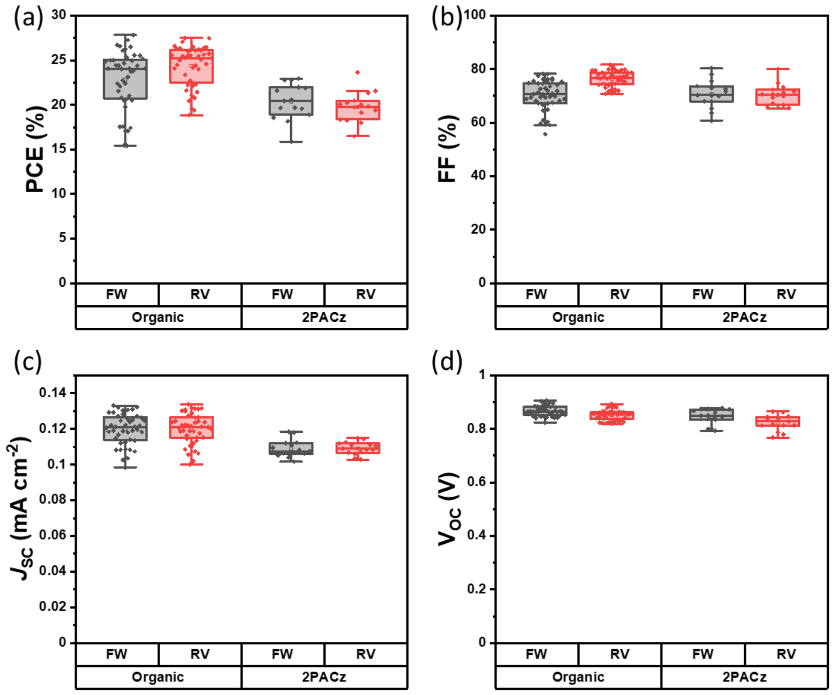

Figure S6. 1 Sun photovoltaic performance parameters of (a) PCE, (b) fill factor, (c) J_{SC} (d) V_{OC} for organic (poly-TPD/PFN) and 2PACz HEL-based photovoltaic devices.

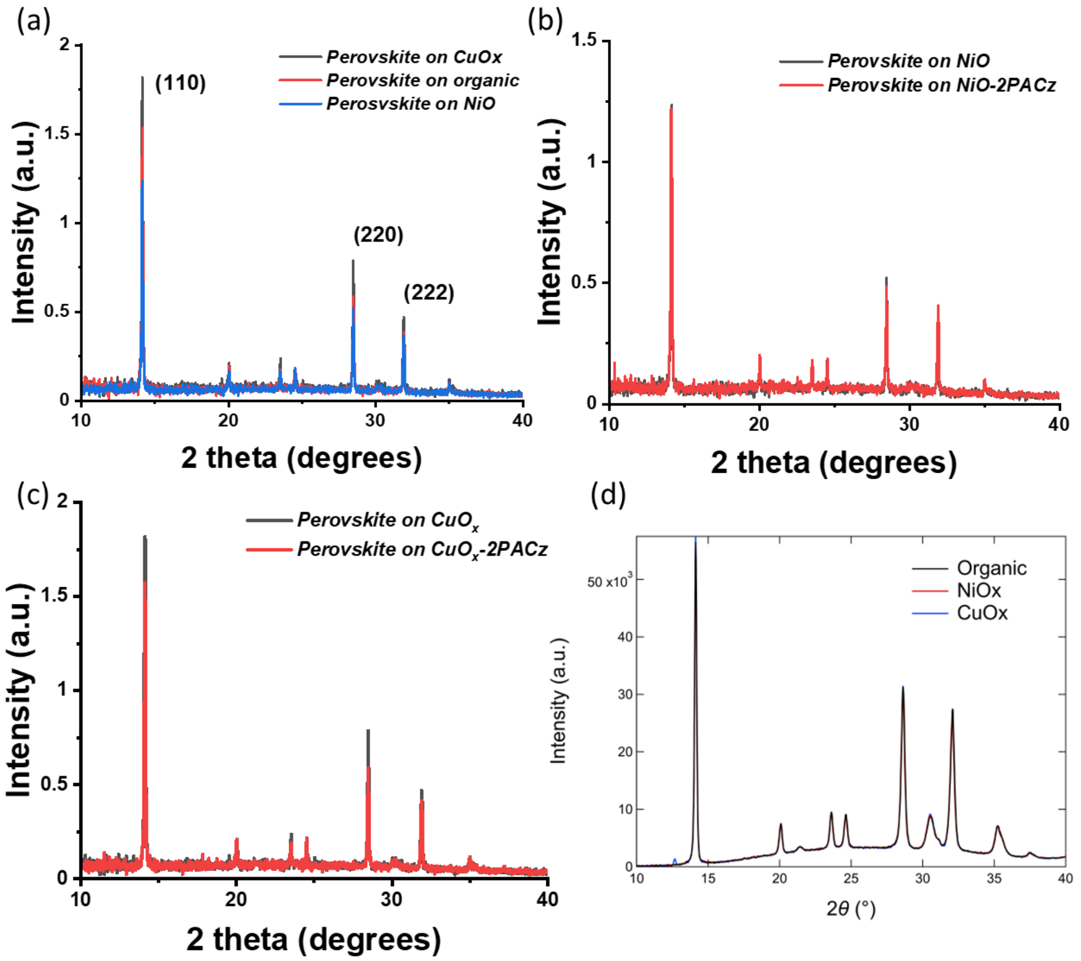

Figure S7. XRD pattern for (a) perovskite films grown on organic (poly-TPD), NiO and CuO_x HELs. (b) perovskite films grown on NiO and 2PACz passivated NiO HELs. (c) perovskite films grown on CuO_x and 2PACz passivated CuO_x HELs. (d) Cu Kα XRD pattern converted from 2D GIWAXS pattern.

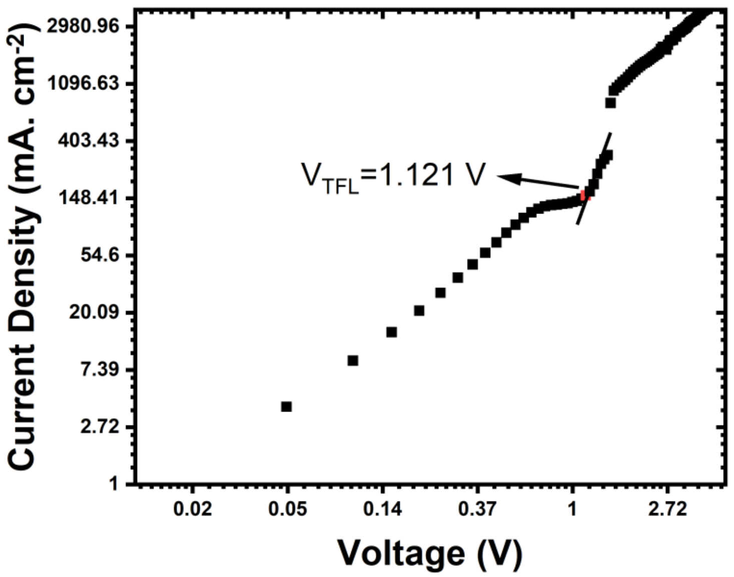

Figure S8. Space-charge limit current model from pristine 2PACz based hole only devices.

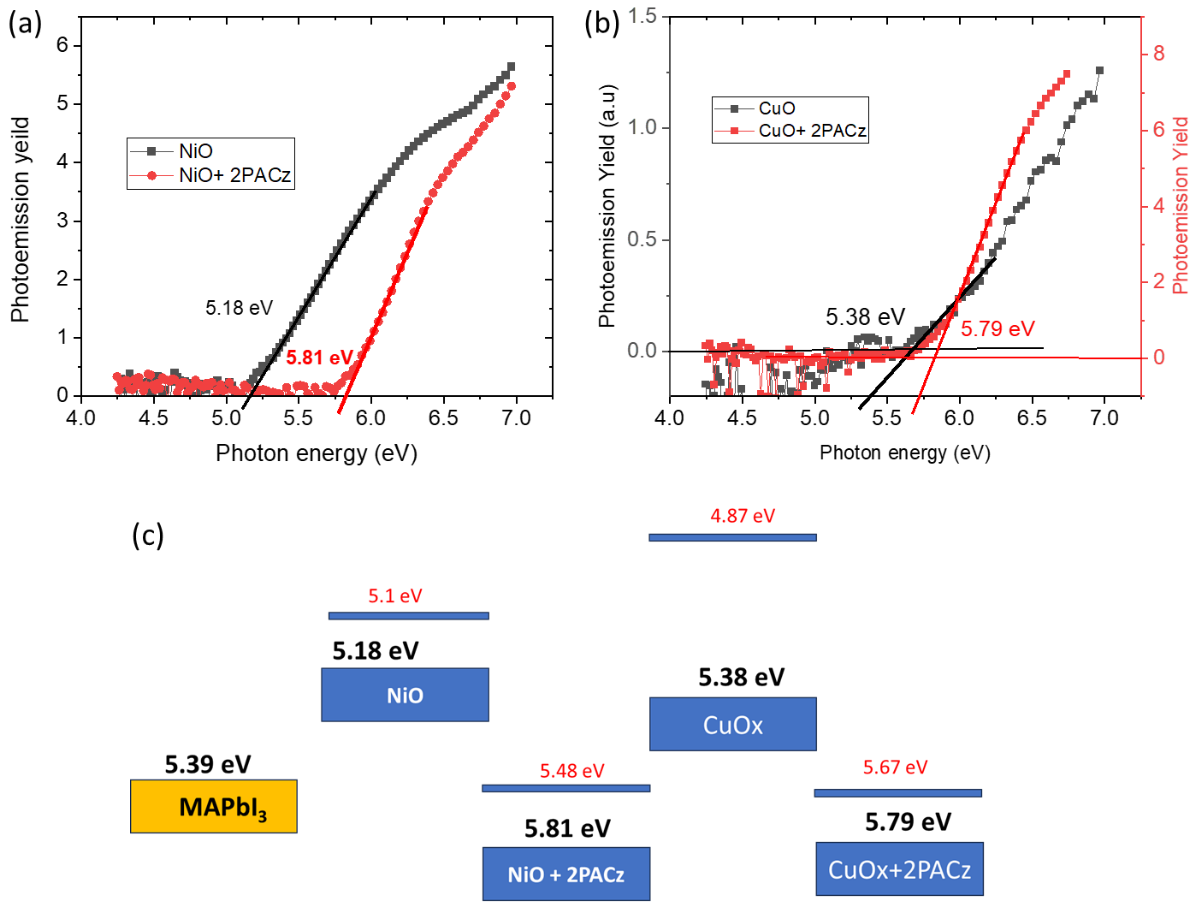

Figure S9. (a) APS spectra of NiO and NiO/2PACz films (b) APS spectra of CuO_X and CuO_X/2PACz films (c) Valence band and work function of metal oxide films and 2PACz modified films. The valence band energy level of $MAPbI_3$ is also shown.

Reference

1. Hsiao, K. C. *et al.* Enhancing Efficiency and Stability of Hot Casting p-i-n Perovskite Solar Cell via Dipolar Ion Passivation. *ACS Appl Energy Mater* **2**, 4821–4832 (2019).
2. D., J. V. Electronic processes in ionic crystals (Mott, N. F.; Gurney, R. W.). *J Chem Educ* **42**, A692 (1965).
3. Lazanas, A. C. & Prodromidis, M. I. Electrochemical Impedance Spectroscopy–A Tutorial. *ACS Measurement Science Au* **3**, 162–193 (2023).
4. von Hauff, E. & Klotz, D. Impedance spectroscopy for perovskite solar cells: characterisation, analysis, and diagnosis. *J Mater Chem C Mater* **10**, 742–761 (2022).
